# Analysis of the shape of the T-wave in congenital long-QT syndrome type 3 by geometric morphometrics

**DOI:** 10.1038/s41598-021-91346-5

**Published:** 2021-06-07

**Authors:** Hitoshi Horigome, Yasuhiro Ishikawa, Kazuhiro Takahashi, Masao Yoshinaga, Naokata Sumitomo

**Affiliations:** 1 Department of Child Health, Faculty of Medicine, University of Tsukuba, Tsukuba, Ibaraki Japan; 2 Ishikawa Medical Clinic, Internal Medicine, Saitama, Japan; 3 Department of Pediatrics, Nagara Medical Center, Gifu, Japan; 4 Department of Pediatrics, National Hospital Organization Kagoshima Medical Center, Kagoshima, Japan; 5 Department of Pediatric Cardiology, Saitama Medical University International Medical Center, Hidaka, Japan

**Keywords:** Cardiology, Medical research

## Abstract

The characteristic shape of the T-wave in congenital long-QT syndrome type 3 (LQTS3) is considered a late-onset T-wave. We analyzed the difference in the shapes of the T-waves of V5 in the electrocardiograms (ECGs) of LQTS3 cases and normal subjects using generalized Procrustes analysis. The J and Q points of LQTS3 cases are shifted to the upper left compared to those of normal subjects. SdFmax is the point on the ECG where the second derivative is first maximized. SdFmax is the point where the change in the slope of the ascending limb of the T-wave is maximized. SdFmax in LQTS3 cases is shifted to the lower right compared to normal subjects. The interval from J to SdFmax in LQTS3 cases is expanded compared with that of normal subjects. From principal component analysis of the Procrustes mean shape of the T-wave landmarks, the second principal component shows a shift of SdFmax to the lower right. These results can quantitatively explain why the T-wave of LQTS3 cases looks like a late-onset T-wave. After being fitted to a multivariate logistic regression model, LQTS3 cases and normal subjects can be distinguished by the second independent component.

## Introduction

Abnormalities in the shape of the T-wave and QT prolongation are often observed in congenital long-QT syndrome (LQTS). In recent years, it has been found that the cause of LQTS is abnormalities in myocardial ion channels. The LQTS3 model^1^ was induced by sea anemone toxin, a Na+ channel inactivation blocker. It prolonged epicardial, M cell, and endocardial action potential durations (APDs) by prolonging phase 2, producing a long-ST segment, late-onset T-wave, and markedly prolonged QT interval. Mutations in the ion channels have been shown to indicate the characteristic shape of T-waves to a certain degree^2,3^. However, the classification of the shapes of T-waves in LQTSs is subjective and intuitive. To date, it has been difficult to determine whether the shapes of T-waves can be analyzed quantitatively. Recently, a method called “geometric morphometrics” or “statistical shape analysis” has been established as a tool for a quantitative analysis of the shape to determine the transformation and mutation of a creature. LQTS type 3 (LQTS3) has a third of the frequency of the 15 currently known forms of LQTS, and a “late-onset T-wave” is said to be the feature of the shape of the T-wave of LQTS3. We analyzed the possibility of distinguishing LQTS3 subjects from normal subjects by the difference in the shapes of the T-waves using methodology from statistical shape analysis^4^ (geometric morphometrics) ^5–7^. Since the “late-onset T-wave” of LQTS3 in our cases was mainly observed in the lead V5 of the electrocardiogram (ECG), we analyzed only the lead V5 in the ECG. Initially, we need to describe the shape by locating a finite number of points, called landmarks, on each specimen. A landmark is a point of correspondence on each object between and within populations^4,5^. A three-dimensional curve is defined by the curvature and the torsion, while a two-dimensional curve can be defined by only the curvature (according to the Frenet-Serret formulas)^8,9^.

Intuitively, the curvature describes, for any part of a curve, how much the curve direction changes over a small distance (e.g., an angle in rad/m, therefore, the unit is a nonphysical unit.), so it is a measure of the instantaneous rate of change of direction of a point that moves on the curve: the larger the curvature is, the larger this rate of change.

The curvature can be calculated from the first and second derivatives as follows: if the plane curve is given in Cartesian coordinates as y(x), then the curvature is κ = *y′′/*(1 + *y′*^2^)^3*/*2^, where *y′* = *dy/dx, and y′′* = *d*^2^*y/dx*^2^.

Although the curvature and the second derivative are strictly different, when the slope of the graph (that is, the derivative of the function, y′) is small, the signed curvature is approximated well by the second derivative (y″)^9^. Therefore, the points at which the maximum and minimum values of the first and second derivatives occur are considered important landmarks.

## Methods

### Subjects

We studied 12 patients (age 17*.*2 ± 13*.*3 years; 7 males, 5 females) with genetically confirmed LQTS3 (by E1784k gene mutation) and 12 age-matched healthy control subjects free from cardiovascular diseases and medications with electrophysiological effects. The study protocol was approved by the Ethics Committee of the University Hospital of Tsukuba (Ibaraki, Japan), and informed consent was obtained from each patient or parents if the patient was younger than 15 years. The study was performed in accordance with the ethical standards as laid down in the 1964 Declaration of Helsinki and its later amendments or comparable ethical standards. this can be included in the methods section^10^.

Clinical characteristics of the participants in the LQTS3 group. Nine of the 12 patients had late-onset T-waves. Three of the 12 patients had asymmetry, as presented in Table 1. Six of the 12 patients were taking mexiletine (Table 1). Discontinuation of the drugs during the study period was considered risky for these 6 patients. The remaining 6 patients were not on anti-arrhythmic drugs when the ECGs were recorded.

**Table 1:** Clinical characteristics of patients with LQTS3 (E1784k gene mutation).

Case no	Shape of V5	Sex	Age	Symptoms	Mexiletine	Heart rate (bpm)
1	Asymmetric	M	13	Syncope	On	53
2	Asymmetric	M	11	None	Off	76
3	Asymmetric	F	6	Convulsions	On	80
4	Late-onset	M	13	Syncope	On	66
5	Late-onset	F	15	None	On	48
6	Late-onset	F	12	Syncope	On	60
7	Late-onset	M	9	None	On	83
8	Late-onset	F	22	None	Off	59
9	Late-onset	F	59	None	Off	54
10	Late-onset	M	10	None	Off	62
11	Late-onset	M	19	None	Off	51
12	Late-onset	M	17	None	Off	47

### Sampling of ECG data

#### Data acquisition

The ECG was collected using active electrodes to avoid AC power in the shield room. It is equivalent to these conditions to take a noninvasive fetal electrocardiogram^11–13^.

The ECG was recorded as time series data using an ECG amplifier (Polymate AP1532; TEAC; Tokyo). The time constant was set at 3.2 seconds. Therefore, the low cutoff frequency was 0.05 Hz or less. At the same time, a 50 Hz notch filter was used to suppress the interference of the 50 Hz power line. Signals were recorded from 10 channels using 20 active electrodes (TEAC; Tokyo). Channel 1 was set as lead I; channel 2 as lead II; channel 3 as lead III; channels 4 to 9 as bipolar leads from chest to left leg, each corresponding to C1 to C6 of the conventional 12-lead ECG; and channel 10 as 4C9, representing a bipolar lead from the fourth intercostal space on the left spine border of the back to the fourth intercostal space at the left sternal border of the forechest. In each subject, the recorded data were digitized online with an A/D converter (EC-2360; Elmec; Tokyo, Japan) at a sampling rate of 1000 Hz and saved in a notebook computer as a data file for future analysis. The data of C1 to C6 were converted into V1 to V6 using the following formula to produce ECG images: Vi = Ci+(II + III)/3, where i = 1 to 6^14,15^.

The unit of our sampling data is 1.0 at 1 millivolts and 1.0 at 1 millisecond. In the description below, the y-coordinate refers to the signal amplitude (measured in millivolts), and the x-axis refers to the time (milliseconds).

#### Data subsampling

The 9 landmarks below were measured with R language^16^ software for 10 consecutive beats in lead V5 at each ECG.

**Figure 1:**
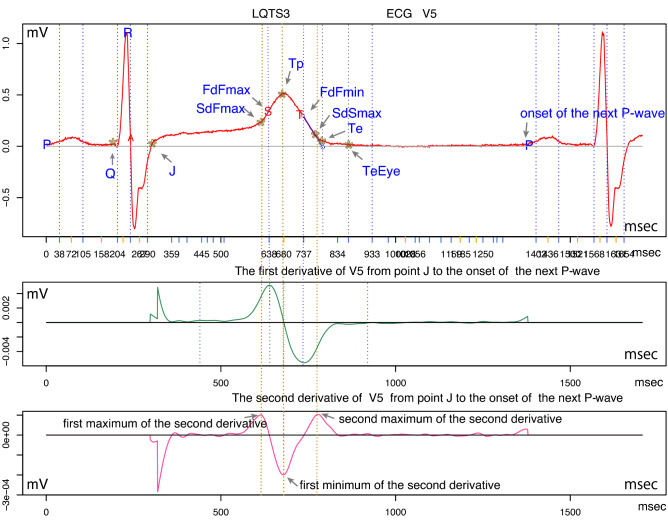
The top panel shows V5 of an ECG (red). The middle panel shows the first derivative (green). The bottom panel shows the second derivative (pink). Abbreviations are described in the text. R version 4.0.2 URL https://www.R-project.org/.

In Figure 1, the top panel shows V5 of the ECG. The middle panel shows the first derivative. The lower panel shows the second derivative from point J to the next starting point of the P-wave. We chose the following nine points in V5 as landmarks:

1. The starting point of the Q-wave, where *Q* is (x, y) = (0, 0).
2. The coordinates of point J are *J*, and the starting point of *J* is *Q*. In what follows, *Q* is a starting point.
3. The coordinates of V5 where the first local maximum of the second derivative occurs (SdFmax).
4. The coordinates of V5 where the first local maximum of the first derivative occurs (FdFmax).
5. The coordinates of the peak of the T-wave (Tp).
6. The coordinates of V5 where the first local minimum of the first derivative occurs (FdFmin).
7. The coordinates of V5 where the second local maximum of the second derivative occurs (SdSmax).
8. The x-coordinate indicating that a tangent from the FdFmin point crosses a baseline (Tend) and the y-coordinate indicating the Tend of V5 (Te).
9. The visual end of the T-wave, y = 0 (TeEye).

The analysis procedure, the method of determining each point and the definitions are given below.

(Please also refer to the software instruction manual in the supplementary material.)

1. Launch the software and open the data file. (Software instruction manual Figure Manual S1.)
2. Select the channel to measure. (Software instruction manual Figure Manual S2.)
3. Determine how many beats can be seen, as in Figure Manual S3, to calculate a rough RR interval to display one heartbeat.
4. On the screen where V5 of one beat is displayed, determine the RR interval and the starting point of the P wave with the naked eye by clicking the mouse. (Software instruction manual Figure Manual S4.)
5. Select the starting points of the P wave and the P wave of the next heartbeat with the mouse, and the interval of these two points is then linearly converted so that these two points become 0 (origin).This can be repeated until the correction is deemed appropriate. (Software instruction manual Figure Manual S5.)
  Figure Manual S5 of the supplementary procedure manual shows a figure with a baseline drawn with the starting point of the P wave (origin) and that of the next P wave as zero (origin) is shown.
6. The vertical dotted blue line in the graph (top panel of Fig. 1, Figure Manual S6 of the supplementary procedure manual) is where the second derivative is zero. The mouse operations are as follows: If the shape of the T -wave is not bifid, Click, in the order of Q, R, Act, FdFmax, FdFmin,TendEye, J, next P, as in Figure Manual 6S.
7. *Q* indicates the starting point of the Q-wave. (x, y) = (0, 0)
  Click with the mouse, on the point *Q* at which the QRS complex first intersects the baseline.
8. The coordinates of point J are *J*, and the starting point of *J* is *Q*. In what follows, *Q* is a starting point. Click with the mouse on the point J at which the QRS complex last intersects the baseline.
  (Ace is an abbreviation for activation time, but it was not involved in this study.)
9. The coordinates of V5 at which the second derivative reaches a local maximum for the first time (SdFmax).
  SdFmax is the point where the orange vertical dashed line passes through the point indicating that the first maximum of the second derivative in the third panel in Figure 1 intersects the ECG of V5 on the top panel.
  The x coordinate of SdFmax can be calculated as the maximum between the point 30 msec from point *J* and the apex of the T-wave in the second derivative.
  SdFmax is not determined by clicking with the mouse.
10. Determine the coordinates of V5 where the first derivative reaches a local maximum for the first time (FdFmax).
  In this study, in the ascending limb near the apex of the T wave of all 24 ECGs, the point on the vertical dotted blue line where the second derivative is 0 that intersects the T wave is only one point. Therefore, FdFmax can be determined with the mouse.
11. The coordinates of the peak of the T-wave (Tp). The x-coordinate of the T-wave peak is obtained from the point closest to the left of FdFmin in the list of points where the first derivative is 0.
12. Determine the coordinates of V5 where the first derivative reaches a local minimum for the first time (FdFmin).
  In this study, in the descending limb near the apex of the T wave of V5 in the ECGs, the point on the vertical dotted blue line where the second derivative is 0 that intersects the T wave is only one point. Therefore, FdFmin can be determined with the mouse.
13. The coordinates of V5 where the second derivative reaches a local maximum for the second time (SdSmax).
  The maximum value of the second derivative between FdFmin and TeEye is calculated to obtain SdSmax.
14. The x-coordinate of the T-wave end is calculated by the tangent method (Tend) as a point at which a tangent from point T (FdFmin) crosses a baseline. The y-coordinate is Tend of V5 (Te).
15. TeEye is the point where the end of the T wave intersects the baseline, and it is obtained visually by clicking with the mouse; y = 0 (TeEye).

The first derivative of V5 is obtained by applying the smoothing spline function of R^16^ to the difference of V5 with a smoothing parameter (spar) of 0.5. The second derivative of V5 is obtained by applying a smoothing spline to this first derivative difference. By default, the smoothing spline function minimizes generalized cross-validation (GCV) and should be minimized unless spar is specified.

The mean of each landmark for each case was analyzed.

The x-coordinates from the distance of *Q* are corrected using √(RR), the revised Bazett correction^17^.

They are not revised about the y-coordinate. The curvature of the T wave is calculated by the formula κ = y″/(1 + y′^2^)^3/2^.

### Analysis

The most common types of statistical tests, parametric, nonparametric, robust and Bayesian versions of the t-test/ANOVA, were carried out with ggstatsplot of the R package^18^. Generalized Procrustes analysis (GPA) was carried out in the shapes of the R package^19^. GPA is a method of optimally registering landmark configurations using translation, rotation and scaling. Goodall’s F test^19^ for mean shape difference, including the permutation and bootstrap test, was performed between 12 normal subjects and 12 LQTS3 subjects. Independent component analysis (ICA) was carried out with the ica packages^20^. Multivariate logistic regression model selection was carried out with bestglm of the R package^21^.

## Results

### Results of ECG measurement

**Figure 2:**
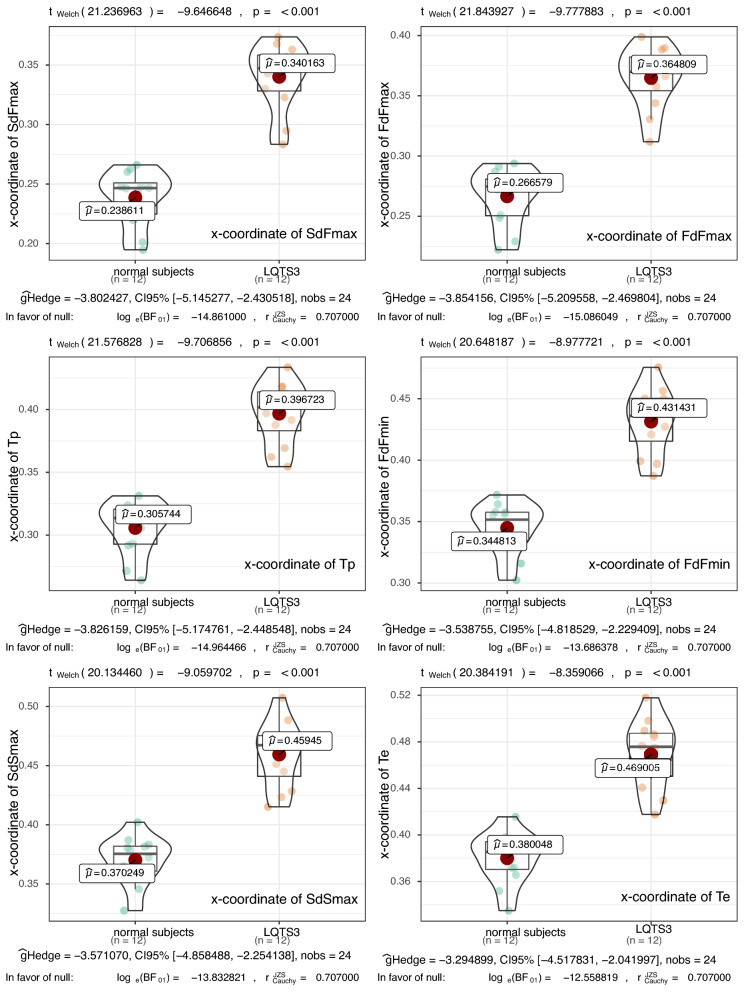
Violin plots for comparing the landmarks of the x-coordinates (SdFmax, FdFmax, Tp, FdFmin, SdSmax and Te) between the normal subjects and the LQTS3 patients. The mean values of the x-coordinate landmarks were greater in the LQTS3 patients than in the normal subjects. The *p* values of the landmarks of the x-coordinates are less than 0.001. Each Bayes factor shows decisive evidence for rejecting our expected null hypothesis. R version 4.0.2 URL https://www.R-project.org/.

Figure 2 shows violin plots for comparing the landmarks of the x-coordinates (SdFmax, FdFmax, Tp, FdFmin, SdSmax and Te) between normal subjects and LQTS3 subjects. The expected null hypothesis in our case is that the values between the two groups do not differ. The mean values of the x-coordinate landmarks were greater for LQTS3 than for normal subjects. The *p* values of the landmarks of the x-coordinates were less than 0.001. The difference between the mean values of the two groups was statistically significant. The Bayes Factor^22^ values log_e_(BF01)^18^ were less than − 12. The Bayes factors also showed decisive evidence in favor of the alternate hypothesis.

The mean values of the y-coordinate landmarks were higher in normal subjects than in LQTS3 subjects, excluding Te. (The figure is not shown.) The *p* value of the y-coordinate of Te was 0.050011. The Bayes factor of the y-coordinate of Te was negligible. The *p* values of the other y-coordinate landmarks were less than 0.01. The Bayes factor of the y-coordinate of SdFmax was − 2.407 and was thought to be strong evidence in favor of the alternate hypothesis. The Bayes factors of the other y-coordinate landmarks were between − 2.05 and − 1.66 and were thought to be substantial evidence for rejecting our expected null hypothesis.

**Figure 3:**
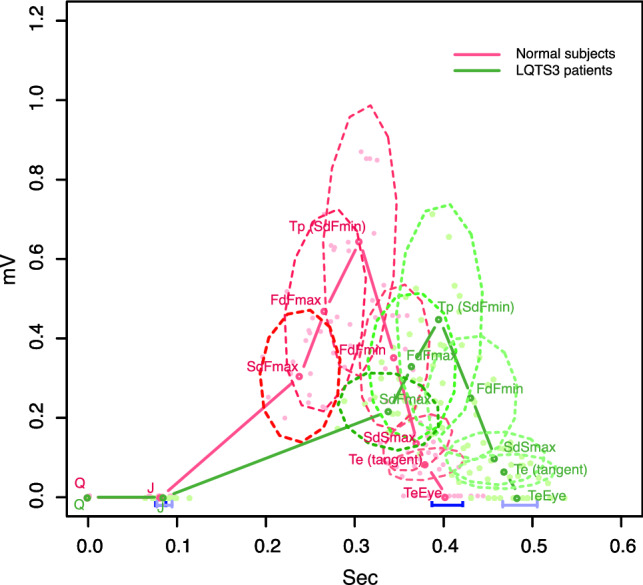
Simply averaged shapes of 9 landmarks of the 12 normal subjects (red) and 12 LQTS3 patients (green). Q is zero at the starting point. The x-coordinates of the landmarks were revised by Bazett correction. The abbreviations in the figure are defined in the main text of this paper. The 95% confidence intervals of the J point and TeEye are indicated by solid blue horizontal lines. The dashed ovals indicate the 95% confidence ellipses for the other landmarks. The corresponding 95% confidence ellipses for the landmarks do not overlap. The pale pink dots are the landmarks of the normal subjects, and the pale green dots are the landmarks of the LQTS3 patients. R version 4.0.2 URL https://www.R-project.org/.

Figure 3 shows the simply averaged shape from the data of 9 landmarks of the 12 normal subjects (red) and 12 LQTS3 cases (green) without translation, rescaling or rotation. *Q* is a zero point at the starting point. The x-coordinates of the landmarks were corrected using the Bazett formula^17^. The 95% confidence interval is indicated by a solid blue horizontal line at *J* and TeEye. The ovals with dashed lines indicate 95% confidence ellipses for the other landmarks. The red 95% confidence ellipse indicates normal subjects, and the green ellipse indicates LQTS3 subjects. The 95% confidence ellipses of the landmarks in the normal subjects and those of the corresponding landmarks of LQTS3 do not overlap with each other. The 95% confidence ellipse of SdFmax of the normal subjects is long in the vertical direction, but that of LQTS3 is exceptionally long in the horizontal direction. However, the 95% confidence ellipses of FdFmax, Tp and FdFmin of the two groups are long in the vertical direction.

In Supplimantal Information, there was no discrepancy between the x-coordinates of SdFmax, SdFmin and SdSmax of LQTS3 and those of the curvature of the T wave.

(Please also refer to SupplementaryInformation-curvature-2ndderivative-12casesLQTS3.xlsx).

### Results of generalized Procrustes analysis

**Figure 4:**
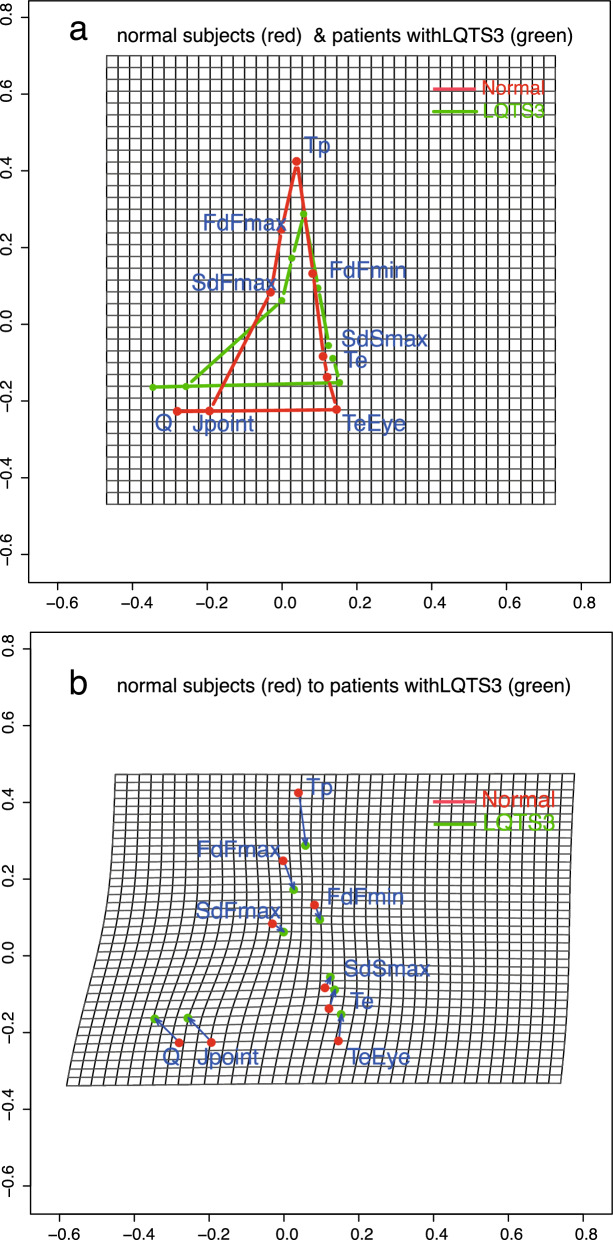
(**a**) Shows the mean shape of the T-wave in the normal subjects (red) and the mean shape of the T-wave in the LQTS3 patients (green) by full GPA. (**b**) Shows thin-plate spline transformation grids to minimize the mixed energy from the normal mean shape (red) to the LQTS3 patient mean shape (green), with blue arrows drawn from the normal subject mean (red) to the LQTS3 patient mean (green). The shape changes have not been magnified. R version 4.0.2 URL https://www.R-project.org/.

GPA was carried out in shapes in the R package^19^. GPA is defined as a method involving translating, rescaling and rotating configurations relative to each other to minimize the total sum of squares^19^. Panel **a** of Figure 4 shows the normal subject (red) mean shape registered to the LQTS3 subject (green) mean shape of the T-wave registered by full GPA. The degree of inclination of the T wave is maximal at the point where the second derivative reaches a local maximum for the first time (SdFmax). SdFmax is the first point which the degree of bending of the T wave is maximized. The SdFmax of LQTS3 subjects (green) was slightly displaced to the lower right compared with that of normal subjects (red). The interval from *J* to SdFmax for LQTS3 subjects (green) was expanded compared with that of normal subjects (red). The slope of the ascending limb of the T-wave becomes largest at the point where the first derivative reaches a local maximum for the first time (FdFmax). Both FdFmax and Tp (peak of the T-wave) of LQTS3 (green) were pulled downward. The displacements of these x-coordinates were only slightly rightward. The amplitudes of LQTS3 subjects were smaller than those of normal subjects. FdFmin is the coordinate at which the slope of the descending limb of the T-wave is minimized. There was not much difference between LQTS3 subjects (FdFmin) and normal subjects. SdSmax is the coordinate of V5 at which the second derivative reaches a local maximum for the second time. SdSmax is the point where the change in inclination of the T wave on the descending limb is maximal. The SdSmax of LQTS3 subjects (green), compared with that of normal subjects (red), is pulled to the right and upward slightly. Both Te and TeEye show an upper right displacement. Comparing normal subjects with LQTS3 subjects, it was observed that the distance between *J* and SdFmax increased and the amplitude of the T-wave decreased. Panel **b** of Figure 4 shows a thin-plate spline transformation grid to minimize the mixed energy from the normal subject mean (red) to the LQTS3 mean (green). A blue arrow is drawn from the normal subject mean shape (red) to the mean shape of LQTS3 subjects (green). The shape change has not been magnified. The space from *J* to SdFmax is stretched. The space near the apex of the T-wave is compressed downward, and the space near the end point of the T-wave is displaced upward.

### Test for the mean shape difference

Goodall’s F test^19^ for the mean shape difference, including the permutation and bootstrap test, was performed between the 12 normal subjects and 12 LQTS3 subjects. From the output of the two independent samples for Goodall’s F test, the tabular *p* value= 0, the permutation (10000 iterations) *p* value is 0.0004 and the bootstrap (10000 iterations) *p* value is 0.0003. There was a significant difference between the mean shape of normal subjects and the mean shape of LQTS3 subjects in Goodall's F test.

### Results of shape PCA and ICA

**Figure 5:**
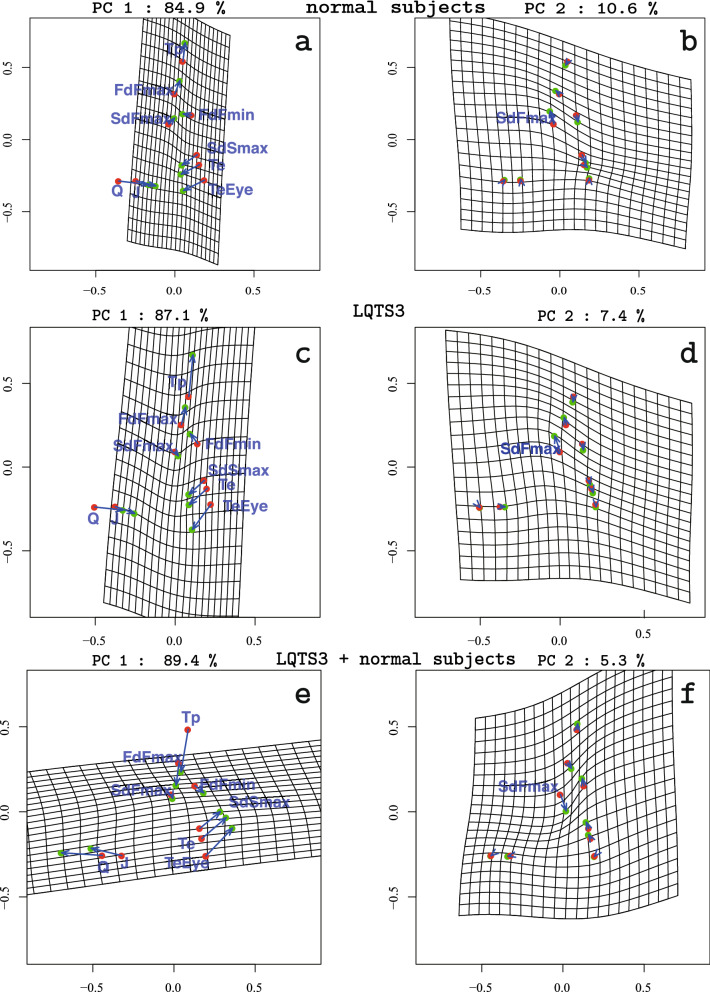
Changes in PC1 and PC2 in the normal subjects (**a**, **b**), patients with LQTS3 (**c**, **d**) and a combination of the two groups of subjects (**e**, **f**). A square grid is drawn on the mean shape (red points) and deformed using a pair of thin-plate splines to an icon (green points, mean + 1 ∗ standard deviation) along each PC (indicated by the blue vector from the mean to the icon). PC1 of the normal subjects (**a**) and PC1 of the LQTS3 patients (**c**) show that Q, J, SdSmax, Te and TeEye are attracted to the vertical center and narrowed. FdFmax and Tp are displaced upwards. The SdFmax of PC2 of the normal subjects (**b**) and that of the LQTS3 patients (**d**) is displaced to the upper left. In the case of the combination of the two types, PC1 (**e**) for the Procrustes mean shape shows the displacement to the left of Q and J. FdFmax and Tp are displaced downward. SdSmax, Te, and TeEye are pulled to the upper right. There are almost no changes in SdFmax and FdFmin. PC2 (**f**) for the Procrustes mean shape shows a displacement of SdFmax to the lower right. R version 4.0.2 URL https://www.R-project.org/.

**Figure 6:**
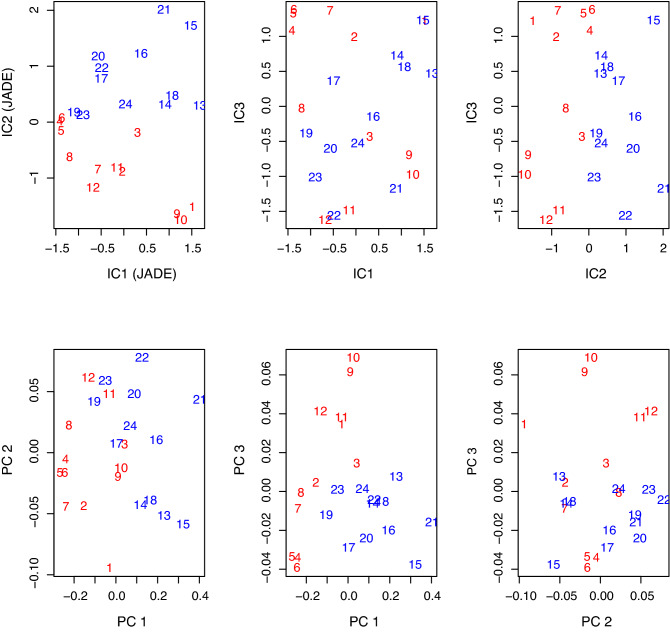
Plot of the first three IC scores (top row) and the first three PC scores (bottom row) for a combination of the landmark data. The red numbers indicate normal subjects, and the blue numbers indicate LQTS3 patients. IC2 yields quite good separation between normal subjects and LQTS3 patients. R version 4.0.2 URL https://www.R-project.org/.

GPA was performed on normal subject landmarks alone, LQTS3 landmarks alone and the combination of the two types (normal subjects and LQTS3) of data. Figure 5 displays the change in the principal components of normal subjects alone (panel **a**, panel **b**), LQTS3 alone (panel **c**, panel **d**) and the combination of the data (panel **e**, panel **f**). The left column of Figure 5 shows PC1, and the right column of Figure 5 shows PC2. A square grid is drawn on the mean shape (red points) and deformed using a pair of thin-plate splines to an icon (green points, c = 1∗standard deviations) along each PC (indicated by a blue vector from the mean to the icon). Each PC1 of normal subjects (panel **a**) and LQTS3 subjects (panel **c**) shows that *Q, J,* SdSmax, Te, and TeEye are attracted to the center and narrowed. FdFmax and Tp are lifted up. Each SdFmax of the PC2 of normal subjects (panel **b**) and LQTS3 subjects (panel **d**) shows displacement to the upper left. In the case of the combination of the two types (normal subjects and LQTS3), PC1 (panel **e**) for the Procrustes mean shape shows a displacement to the left for *Q* and *J*. FdFmax and Tp are displaced downward. Te, SdSmax and TeEye are pulled to the upper right. There is almost no change in SdFmax and FdFmin. The second principal component (PC2) (panel **f**) for the Procrustes mean shape shows a displacement in the lower right for SdFmax. The orientation of the vector of SdFmax is opposite to that of SdFmax in panel **b** and panel **d**. We see that the percentage of variability explained by the first two PCs is 89.4% (panel **e**) and 5.3% (panel **f**), respectively. The result of the principal component analysis (PCA) shows that s (the centroid size)^4,19^ and PC1 yield reasonably good separation of the two groups (the figure is not shown here.). As a further indication of the analysis that can be carried out, we considered ICA, which seeks the most non-Gaussian directions of variability. There are many types of ICA, and we use JADE, which is available in the R library^20^. Figure 6 shows plots of the independent component (IC) scores and PC scores for the T-wave landmark data. We see that the second independent component (IC2) yields quite good separation between normal subjects and LQTS3. The red numbers indicate normal subjects, and the blue numbers indicate LQTS3 subjects.

### Results of the multivariate logistic regression model

Multivariate logistic regression model selection was carried out using bestglm in the R package^21^. Logistic regression is a method for fitting a regression curve, y = f (x), when y is a categorical variable (in this paper, normal subjects or LQTS3 subjects). The typical use of this model is to predict y given a set of predictors (explanatory variables) x. For the objective (response) variable, normal subjects = 1, and LQTS3 = 0. Using five explanatory variables (centroid size s and Riemannian distance ρ^4,19^ to the mean shape and the first three PC scores), we fitted a multivariate logistic regression model for these data. For all combinations of the five explanatory variables, variable selection was performed using the minimization of Akaike's information criterion (AIC) as an indicator. *p* = (exp(689.8 − 995.3 ∗ s− 1307.2 ∗PC1)/(1 + exp(689.8 − 995.3 ∗ s− 1307.2 ∗PC1)) was adopted as the best model (AIC=6). In this equation, normal subjects and LQTS3 subjects can be completely separated. (The figure is not displayed.) Using five explanatory variables (the centroid size s and Riemannian distance ρ to the mean shape and the first three IC scores), we fitted a multivariate logistic regression model for these data. For all combinations of the 5 explanatory variables, variable selection was performed with AIC minimization as a guide. *p* = (exp(72.91 + 748.09 ∗ IC2)/(1 + exp(72.91 + 748.09 ∗ IC2)) was adopted as the best model (AIC=4). Based on the AIC, this expression was the best model. With this equation, normal subjects and LQTS3 subjects can be completely separated.

## Discussion

We analyzed the possibility of distinguishing LQTS3 subjects from normal subjects by the difference in the shapes of T-waves using methodology from statistical shape analysis. We analyzed the difference in the shapes of T-waves using GPA. We found that the *Q* and *J* of LQTS3 subjects appear in the upper left area more than the *Q* and *J* of normal subjects. The curvature and SdFmax are strictly different. The SdFmax of LQTS3 subjects is the point at which the second derivative takes the first maximum value, and the point at which the change in the slope of the T wave is the largest is slightly displaced to the lower right compared to that of normal subjects. The interval from *J* to SdFmax of LQTS3 subjects is expanded compared with that of normal subjects. These two results suggest that the rising point of the T-wave of LQTS3 subjects is later than that of normal subjects and looks like the "late-onset" shape. The shape PCA of the T-wave landmark data shows that a displacement of *Q* and *J* to the left and a displacement of Te and TeEye to the right were observed on PC1 (In Figure 5e). There are almost no changes in SdFmax and FdFmin.

The second PC for the Procrustes mean shape shows a displacement to the lower right for SdFmax (Figure 5f*)*. These results also suggest that a "late-onset T-wave" is the feature of the shape of the T-wave of LQTS3. Shape PCA and ICA were performed to discriminate LQTS3 subjects from normal subjects. Multivariate logistic regression model selection was carried out to discriminate between normal subjects and LQTS3 subjects. We see that s (the centroid size) and PC1 yield reasonably good separation of the two groups. IC2 yields better separation than the results of PCA. Fitting our data to a multivariate logistic regression model, *p* = (exp(72.91 + 748.09 ∗IC2)/(1 + exp(72.91 + 748.09 ∗IC2)) was adopted as the best model (AIC=4). With this equation, normal subjects and LQTS3 subjects can be completely separated.

LQTS3 was intuitively classified as 53% late-onset T-waves, 12% asymmetric T-waves, and 33% overlapping LQT type 1 pattern^3^. In Figure 3, the 95% confidence ellipse of SdFmax in normal subjects is long in the vertical direction, but the 95% confidence ellipse of SdFmax in LQTS3 subjects is exceptionally long in the horizontal direction, which suggests that LQTS3 may contain several patterns other than late-onset.

Choosing the electrodes is an important issue in electrocardiographic analysis.

Fortunately, in this study, "late-onset T-waves" were mainly observed on lead V5.

However, it is generally difficult to find reasonable justifications to choose channels.

We may need to analyze the vector ECG in 3D space to obtain a comprehensive analysis.

The vertical plane (x, y) can be calculated using leads I, II, and III.

By acquiring the horizontal plane (z) vector 4C9, we prepared for future research.

### Limitations

#### This study has several limitations

First, although we made an effort to prevent noise as much as possible, various internal noise signals, such as respiratory movements and muscle contractions, in addition to extrinsic noise, such as electromagnetic waves, can interfere with the ECG recording.

There is no universal method of reducing noise. Noise reduction is always a trade-off between fitness and smoothness^11–13,23^.

Configurational changes in the ECG waveforms are frequently observed after noise reduction and downsampling.

Spline smoothing is a trade-off between bias and variance^24^.

If there is considerable noise, the spline smoothing process for obtaining the first and second derivatives can be significantly affected.

In the first derivative, when the smoothing parameter (spar) was changed from 0.7 to 0.2 by 0.1, weselected spar 0.5, which yielded a good balance between fitness and smoothness.

For each spar, the information entropy^11,25,26^ and root-mean-square error (RMSE) were calculated.

The information entropy and RMSE decreased in spar 0.7 and spar 0.6, Below spar 0.5, the information entropy and RMSE hardly decreased.

On the other hand, smoothness was significantly lost at spar 0.4 or less, so we chose spar 0.5.

Additionally, for the second derivative, generalized cross-validation provided sufficient smoothness (default for the smooth.spline R function^16^), so we did not use the spar.

However, regarding the selection of the smoothing parameters, the information entropy and other parameters should be carefully selected as an evaluation index^25,26^.

In Figure 1, we can visually confirm whether there are discrepancies between the maximum and minimum of the first derivative and the second derivative and the shape of the T wave on the top panel.

In this study, the T wave increases almost monotonically to the apex and decreases monotonically to the end of the T wave.

There is almost no discrepancy between the maximum and minimum of the first derivative and the second derivative and the shape of the T wave. Although the curvature and the second derivative are strictly different, there is no discrepancy between the x-coordinates of the maximum and minimum of the second derivative and the x-coordinates of the curvature of the T wave, which were calculated by the formula κ = y″/(1 + y′^2^)^3/2^.

If there is little noise and there is no large discrepancy between the T–wave of V5 and the first and second derivatives, then the first derivative is small and the curvature can be approximated with the second derivative in our cases.

Second, even in a linear transformation in which the starting point of a P wave and the starting point of the next P wave are zero (origin), a deformation of the T wave is often observed when the fluctuation of the baseline is large.

In approximately 10% of the cases, analysis was abandoned due to significant baseline sway or noise.

Further improvement is required to remove noise from the fluctuation of the baseline.

Third, this study may be criticized for its relatively small sample size.

## Supplementary Information

Supplementary Information 1.

Supplementary Information 2.

## Data Availability

The original R software, and the datasets generated and analyzed during the current study are available from the corresponding author on reasonable request.
