# Publisher Correction: Analysis of the shape of the T-wave in congenital long-QT syndrome type 3 by geometric morphometrics

**DOI:** 10.1038/s41598-021-96118-9

**Published:** 2021-08-19

**Authors:** Hitoshi Horigome, Yasuhiro Ishikawa, Kazuhiro Takahashi, Masao Yoshinaga, Naokata Sumitomo

**Affiliations:** 1 Department of Child Health, Faculty of Medicine, University of Tsukuba, Tsukuba, Ibaraki Japan; 2 Ishikawa Medical Clinic, Internal Medicine, Saitama, Japan; 3 Department of Pediatrics, Nagara Medical Center, Gifu, Japan; 4 Department of Pediatrics, National Hospital Organization Kagoshima Medical Center, Kagoshima, Japan; 5 Department of Pediatric Cardiology, Saitama Medical University International Medical Center, Hidaka, Japan

Correction to: *Scientific Reports* 10.1038/s41598-021-91346-5, published online 07 June 2021

The original version of this Article contained typographical errors.

In Figure 3, the pale pink text, dashes, lines and dots did not display correctly. In addition, the green lines and dots did not display correctly.

The original Figure 3 and accompanying legend appear below.

**Figure 3:**
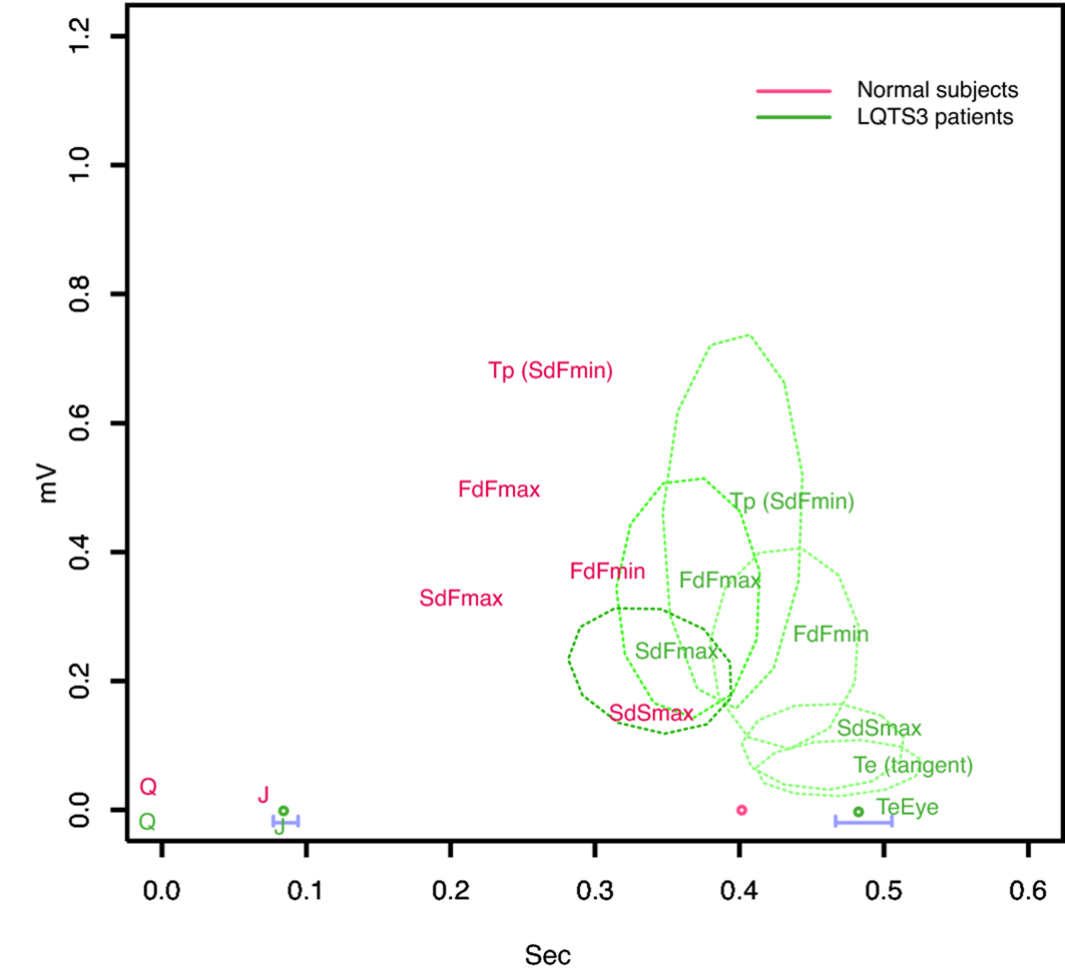
Simply averaged shapes of 9 landmarks of the 12 normal subjects (red) and 12 LQTS3 patients (green). Q is zero at the starting point. The x-coordinates of the landmarks were revised by Bazett correction. The abbreviations in the figure are defined in the main text of this paper. The 95% confidence intervals of the J point and TeEye are indicated by solid blue horizontal lines. The dashed ovals indicate the 95% confidence ellipses for the other landmarks. The corresponding 95% confidence ellipses for the landmarks do not overlap. The pale pink dots are the landmarks of the normal subjects, and the pale green dots are the landmarks of the LQTS3 patients. R version 4.0.2 URL https://www.R-project.org/.

The original Article has been corrected.

