# Supplementary material for: Analysis of the shape of the T-wave in congenital long-QT syndrome type 3 by geometric morphometrics
Source: Sci Rep. 2021 Jun 7;11:11909. doi: 10.1038/s41598-021-91346-5 (PMC8184778; doi:10.1038/s41598-021-91346-5)
Supplement: Supplementary file 1 — Supplementary Information 1. [file 41598_2021_91346_MOESM1_ESM.pdf]

Supplementary Information - manual of R software -

Analysis of the shape of the T-wave in congenital long -QT syndrome type 3 by geometric morphometrics

Hitoshi Horigome[1+], Yasuhiro Ishikawa[2*+], Kazuhiro Takahashi[3], Masao Yoshinaga[4], and Naokata Sumitomo[5]

[1]Department of Child Health, Faculty of Medicine, University of Tsukuba, Tsukuba,Ibaraki,Japan
[2]Ishikawa Medical Clinic, Internal Medicine,Saitama,Japan
[3] Department of Pediatrics, Nagara Medical Center, Gifu, Japan
[4]Department of Pediatrics, National Hospital Organization Kagoshima Medical Center,Kagoshima,Japan
[5]Department of Pediatric Cardiology, Saitama Medical University International Medical Center,Hidaka,Japan
[*]e-mail: wavelet@nifty.ne.jp

1. Select and open data-file.

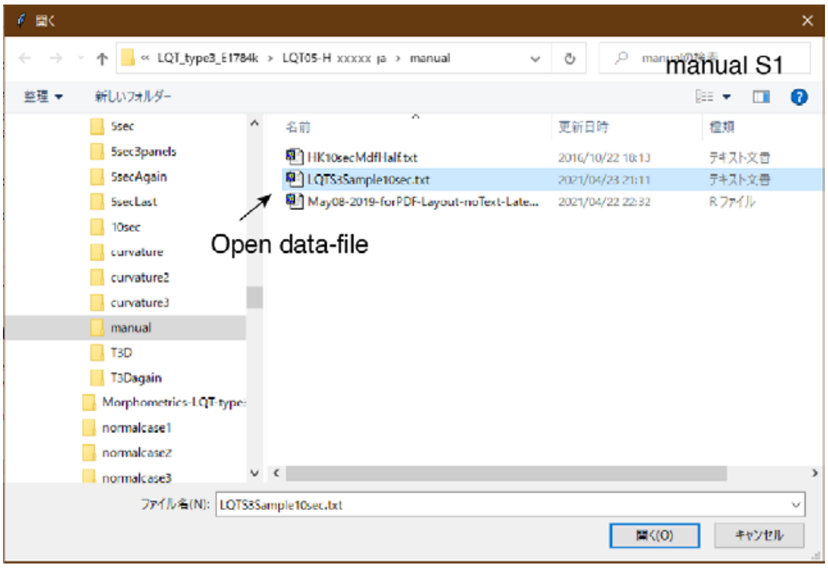

2. Select the channel? 1-10 1 is lead I,2 is lead II,…,4 is V1,…,8 is V5,9 is V6,10 is 4C9

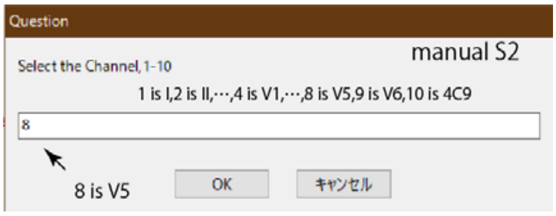

3. How many beats in the Figure? (In this example, the data size is 10 seconds)

 (in order to calculate a rough RR interval to display one beat.)

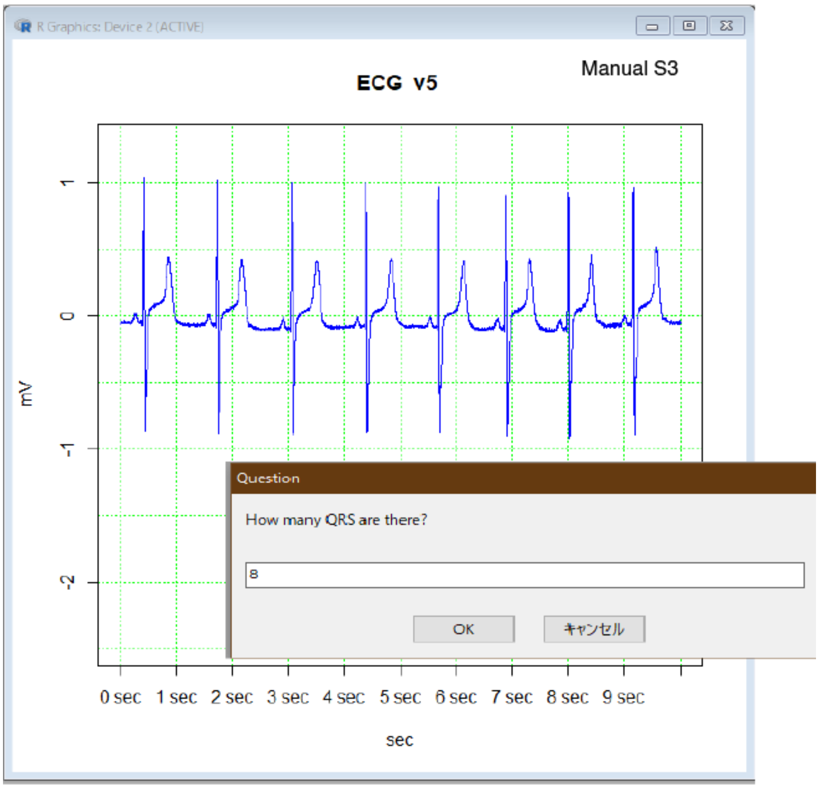

4. Display one heartbeat. The onset of the P wave (pink asterisk No. 1), the R peak of the previous beat (pink asterisk No. 2), the R peak of the next beat (pink asterisk No. 3), Click with the mouse in this order. (to cariculate RR interval.)

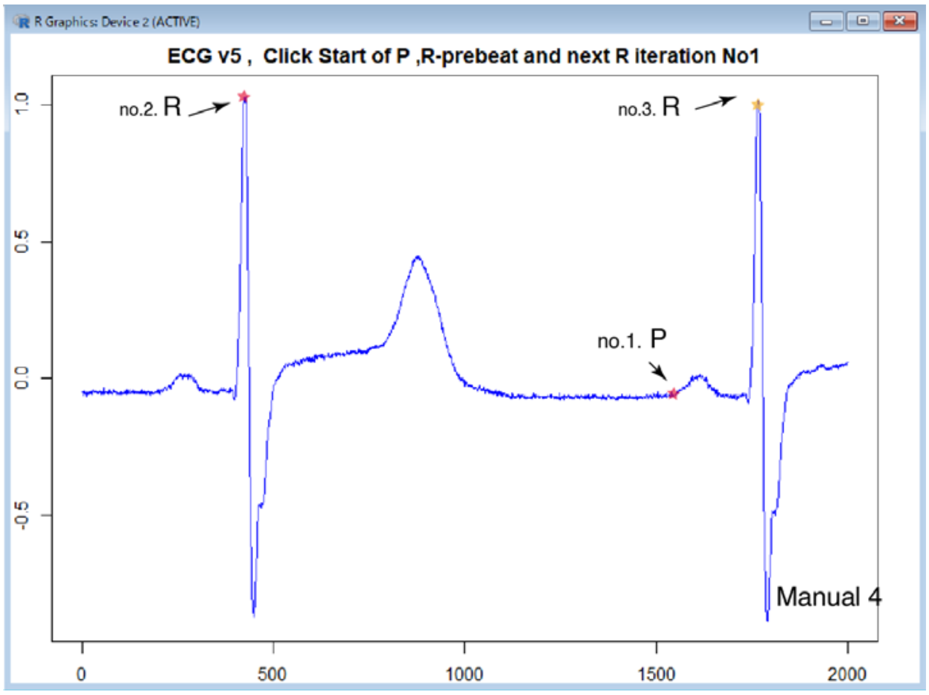

4. Linear transformation with the start point of the P wave as 0 (origin) and the start point of the p wave of the next beat as 0 (origin). Click with the mouse from green asterisk (no.1 P) to next green asterisk (no.2 P). Perform a linear transformation to set the baseline. It can be repeated until the correction is deemed appropriate.

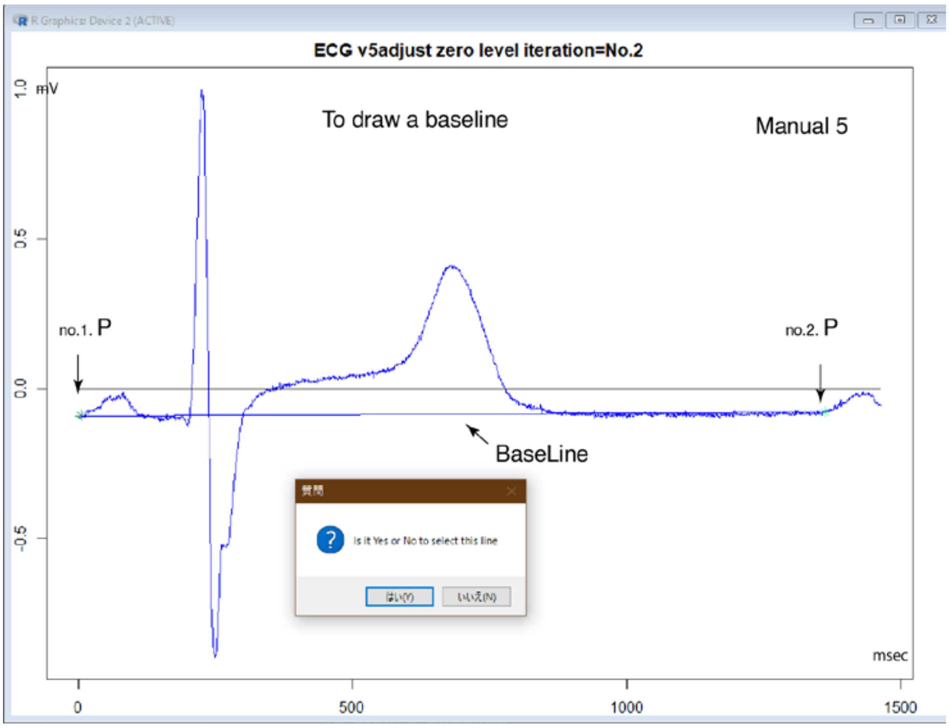

4.The vertical dotted blue line in the graph is where the second derivative is zero. Click, in the order of Q,R,Act, FdFmax, FdFmin, (if shape is not bifid,) TeEye, J, next P

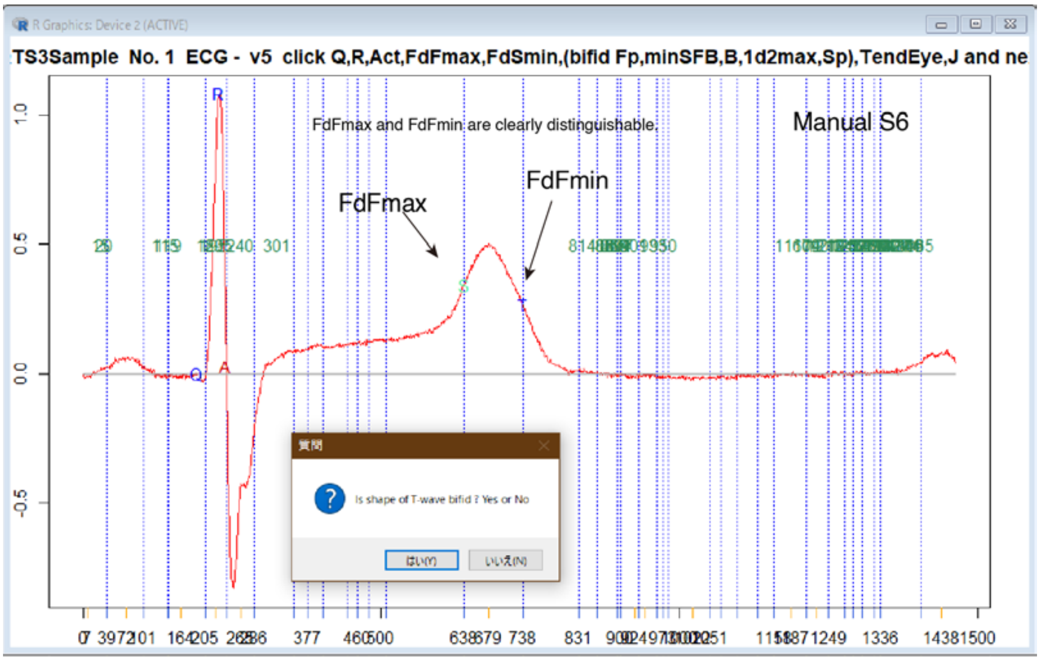

4. You will get this screen after clicking Q, R, Act, FdFmax, FdFmin, TendEye, J, next P, in that order.

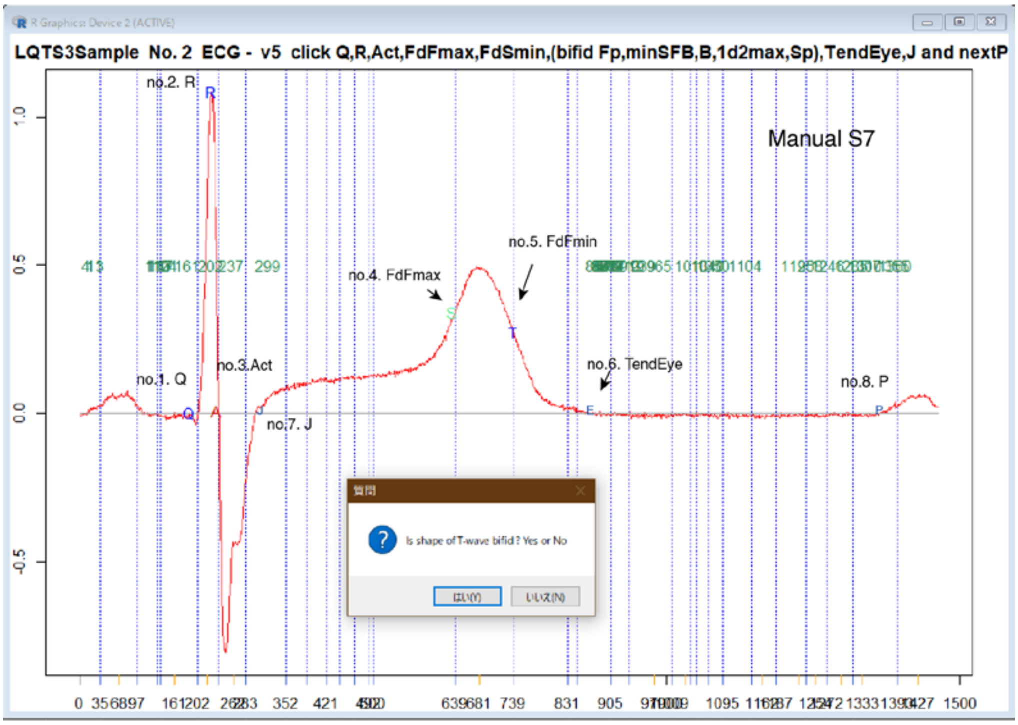

5. Three panels are obtained. The top panel is the V5 of the ECG being analyzed. The second panel is the first derivative. The third panel is the second derivative.

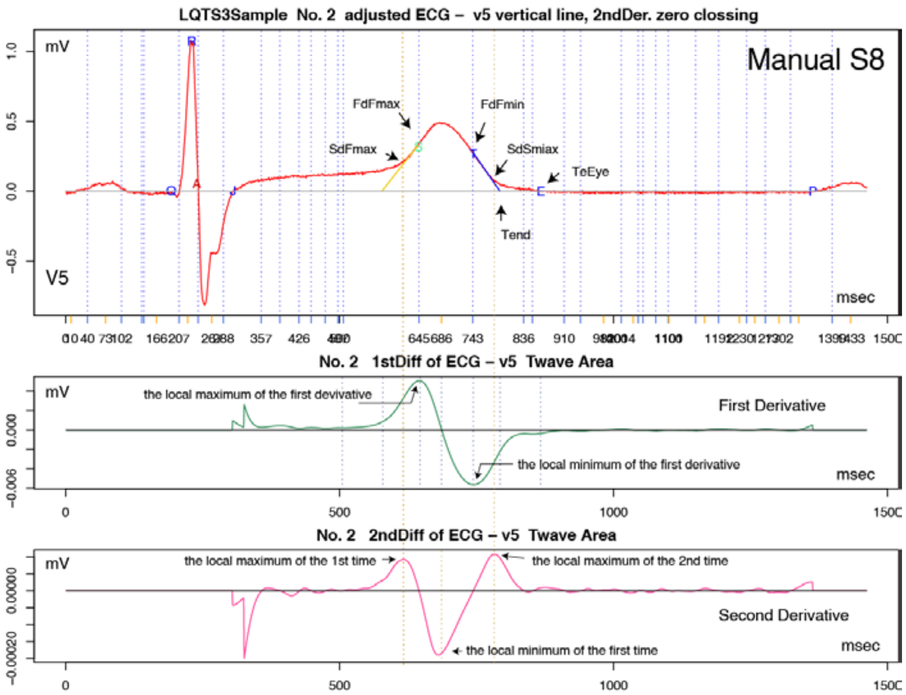

6. A message board will appear asking if you want to approve these operations and use the analysis of this heartbeat as data. If YES, the next heartbeat is displayed. If NO, repeat this heartbeat.

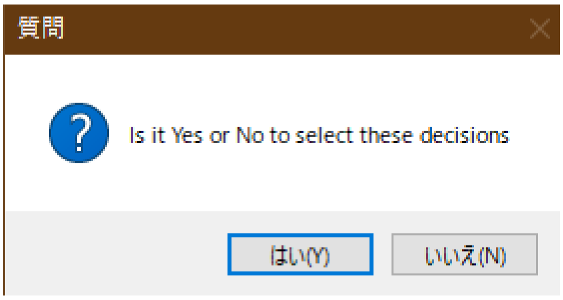

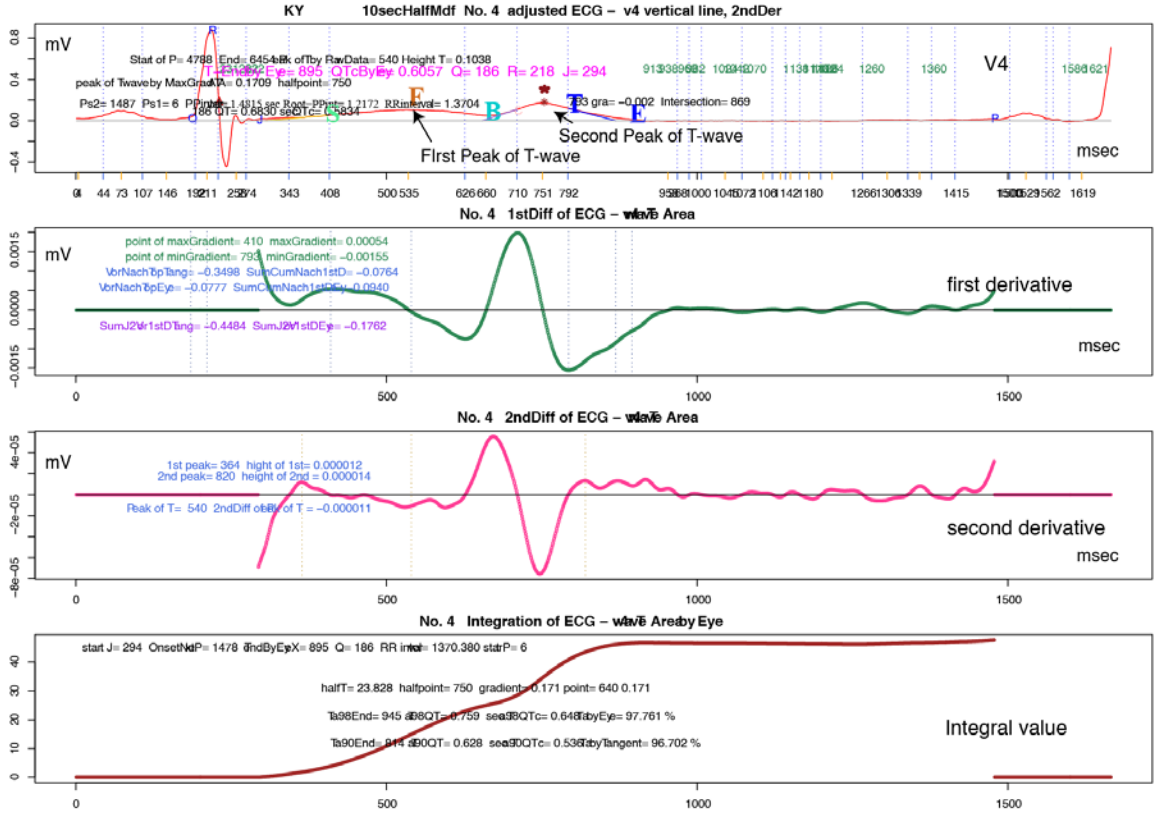

7. A sample of Bifid T-wave (LQT type 2: V4), We are in the middle of our research.

The image of the bifid T wave under study is posted on page 6 of Supplementary-Information.pdf.

We are studying while visually checking the minimum and maximum. In this sample, it seems that further ingenuity is needed for noise reduction, etc.
